# Conjoined twins: A report of four cases

**DOI:** 10.1016/j.ijscr.2020.06.072

**Published:** 2020-06-20

**Authors:** Mohammed Hamada Takrouney, Ibrahim Ali Ibrahim, Hala Saad Abdel-Ghaffar, Ahmed Ibrahim Abdel-Wahhab, Mahmoud Mohamed Mostafa, Wesam Nashat Ali, Mohamed Sayed Abd- Elaal

**Affiliations:** a Pediatric Surgery Department, Faculty of Medicine, Assiut University, Assiut, Egypt; b Anesthesia and Intensive Care Department, Faculty of Medicine, Assiut University, Assiut, Egypt; c Cardiothoracic Surgery Department, Faculty of Medicine, Assiut University, Assiut, Egypt

**Keywords:** Conjoined twins, Parasitic twins, Siame, Omphalopagus, Heteropagus

## Abstract

**Background:**

Conjoined twin is a rarely seen congenital anomaly associated with significant morbidity and mortality. The most common types of conjoined twins are thoracopagus. Conjoined twins are either symmetrical twins or asymmetrical or heteropagus. This report records the successful separation of 2 cases of asymmetrical twins and one symmetrical twin with fused livers and diaphragm and communicating peritoneal cavities.

**Patients and methods:**

This study amended and strictly followed the ethical guidelines of the Helsinki declaration, our study included 4 case reports on conjoined twins (CT), 2 male and three female patients; 3 of them were parasitic and one was conjoined. We operated upon 2 parasitics and one conjoined. The 1st case died preoperatively.

**Results:**

Four living children is our result.

**Conclusion:**

As connections between the bowel and bone of the parasite and the respective organs in the autosite are often absent, the parasite could be excised easily without any effect on the autosite. We recommend the separation of the parasite and the autosite as early as possible. In many cases, surgery results in the death of one or both of the conjoined twins, particularly if they are joined at the head or share a vital organ. Our separated twins are yet the youngest living separated twins.

**Trial registration:**

ClinicalTrials.gov Identifier: NCT03388684

## Highlights

• Conjoined twins are a subset of monozygotic twin gestation.
• They are classified according to the most prominent site of conjunction.
• Surgical separation of conjoined twins is extremely challenging and high risk.
• Our reported omphalopagus had no associated anomalies.
• The presence of associated malformations is unrelated to the site of union.

## 1 Introduction

A conjoined twin is a subset of monozygotic twin gestation [1]. It’s a rare phenomenon with an incidence of 1 in 50,000 to 1:100,000 births with a higher incidence in Africa and Southwest Asia [2]. Based on the terminology proposed by Spencer and colleagues, conjoined twins are classified according to the most prominent site of conjunction into three major groups; *First*, twins with a ventral union; [cephalopagus (head), thoracopagus (Thorax), omphalopagus (Abdomen) and Ischiopagus (pelvis)]. *Second*; twins with a dorsal union; [pygopagus (sacrum), rachipagus (spine, back) and craniopagus (cranium)] and *lastly*; twins with a lateral union that is referred to as parapagus (side). Depending on the aspect of the embryonic disc, the most common types are thoracopagus (19%), [3,4].

Asymmetrical or ”heteropagus” is a set of conjoined twins with major congenital anomalies and attached externally to a relatively normal fetus. The well-developed twin is termed the ”autosite” and the grossly defective fetus is termed the ”parasite” [5]. The incidence of asymmetrical twinning is extremely rare, about 1 per 1–2 million births. In asymmetrical twin sets the most common presentation is omphalopagus joined at either the hypo-gastric or supra-pubic region [6]. Whereas epigastric attachment (Epigastric heteropagus) is sparse with only 45 reports to date [7]. The parasite is dependent on growth on the host, usually no or rarely contains thoracic or dysplastic abdominal organs, both limbs and trunk are the a possible finding. [8]. In almost all cases, antenatal ultrasound can detect the presence of conjoined twins as early as 12 weeks of gestation [9].

The present study work has been reported in line with the PROCESS criteria [10]. It describes an extremely challenging surgical separation of 4 cases of conjoined twins, focusing on surgical aspects, radiologic investigations, anesthetic management, and outcomes.

### 1.1 Case reports

Our study included 4 case reports that included 2 male and three female patients, 3 of them were parasitic and one was conjoined. We operated upon 2 parasitics and one conjoined. The 1st case died preoperatively.

### 1.2 Case number one

**Fig. 1:**
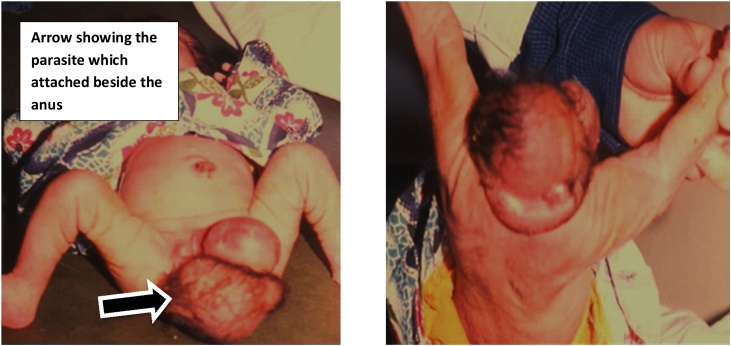
Shows parasitic twins and the parasite was attached to the living one at the perineum posterior to the anus.

A female patient was born at thirty eight weeks. Weight at presentation was 2700 g. The newborn was presented to us complaining of a perineal swelling. The patient Examination revealed parasitic twins. The ”parasite” had an incomplete head and a soft tissue swelling attached to it. The parasite was attached to the living one at the perineum posterior to the anus and shifting it forwards but not attached to the vertebral column (Fig. 1). The patient was passing stool normally. The patient was feverish, without an obvious cause. She was admitted to the neonatal intensive care unit. Unfortunately, the patient died even before doing any investigations because of septicemia.

### 1.3 Case number two

(Epigastric heteropagus)

A male patient was born at thirty nine weeks. birth weight was 3900 g. The Apgar score was normal at the time of presentation and the 1 st and 5th minutes after delivery, respectively. The Male patient was presented a few hours after birth, with a fair general condition and diagnosed clinically as heteropagus.

Examination revealed one complete living twin and the other was a ”parasite”. The patient had an omphalocele major, passing stool normally. The parasite was attached to the epigastric region midway between the xiphisternum and the omphalocele. It had a well-formed lower limb with fixed knees and left foot represented by an appendage, undeveloped right upper limb and rudimentary left one and underdeveloped external genitalia. The patient was subjected to routine laboratory investigations, abdominal ultrasound which revealed the presence of the liver and loops of the intestine in the omphalocele sac.

**Fig. 2:**
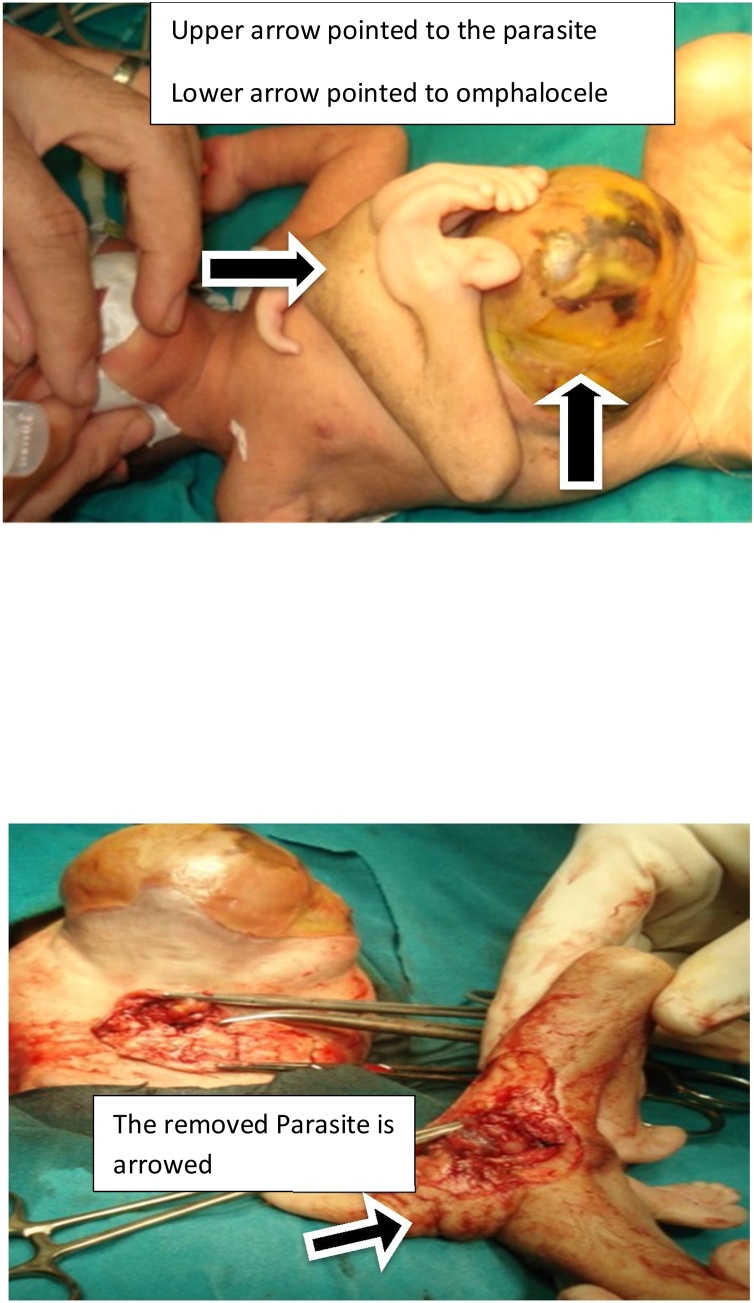
Epigastric heteropagus and the parasite was attached to the epigastric region midway between the xiphisternum and the omphalocele.

Surgery was started by incision at the lower end of the parasite opening its small abdominal cavity. It was attached to the living one by a narrow stalk-like pedicle, enveloped in a peritoneal tube. The contents were feeding vessels, hypoplastic intestinal loops, and soft tissues. The feeding vessels were ligated and the defect in the host abdominal wall was repaired as shown in (Fig. 2). The sac of the omphalocele was excised and the skin only was repaired. The patient passed stool, started oral feeding after 24 h and discharged after one week. The postoperative course passed smoothly.

### 1.4 Case number three

**Fig. 3:**
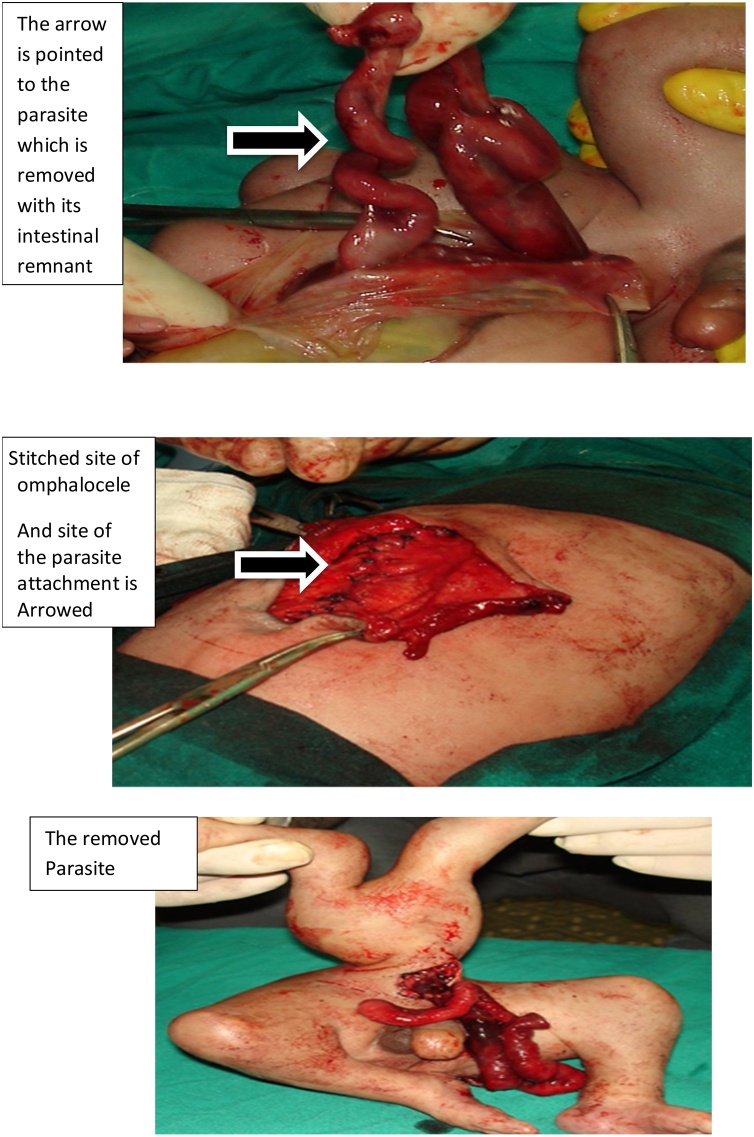
The parasite was connected to the host skin just above the host omphalocele and through it.

A male patient was born at forty weeks. birth weight was 4650 g. The Apgar score was normal at the 1st and 5th minutes after delivery, respectively. The patient was diagnosed prenatally. On examination, the ”parasite” was connected to the host skin just above the host omphalocele and through it. The host was passing meconium normally. The omphalocele contained rudimentary intestine of the parasite without connection to the host GIT of the autosite beside the liver and the intestine of the host. Surgery was done and the parasite was separated from the host and the abdominal wall of the host was repaired with ease with umbilicoplasty (Fig. 3). The patient had a smooth postoperative course, discharged after 5 days of surgery.

### 1.5 Case number four

**Fig. 4:**
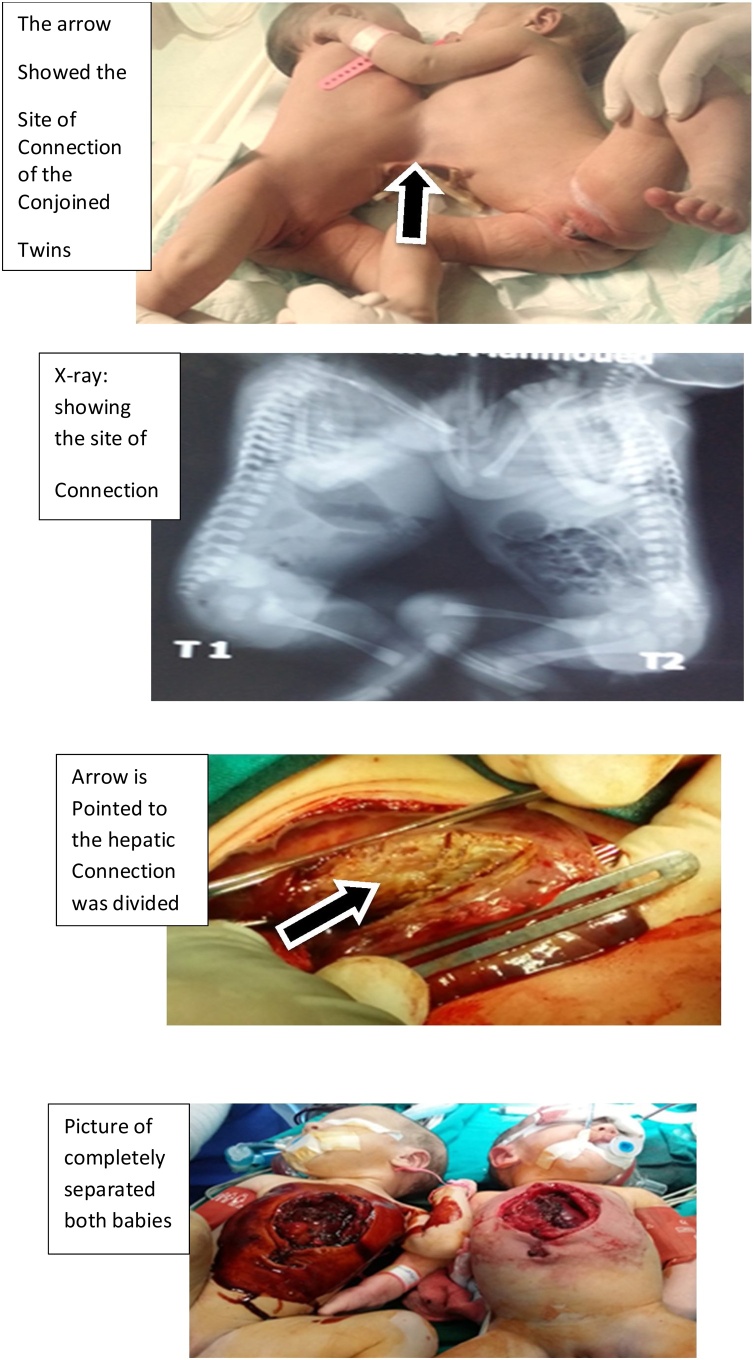
Xiphoomphalopagus conjoined twins conjoined by the sternum and supra-umbilical abdominal wall.

Xiphoomphalopagus conjoined twins presented the same day of delivery at thirty seven weeks. They were diagnosed as twins but discovered to be conjoined during an elective caesarian section. The combined birth weight was 4920 g. The Apgar score was at the 1 st and 5th minutes after delivery, respectively. Clinical examination revealed two living female twins conjoined by the sternum and supra-umbilical abdominal wall, midline conjugation. In the NICU they received feeding and passed meconium normally. Routine laboratory investigations were normal for both. Abdominal ultrasound revealed separate urinary systems with the two livers beside each other. Multi-slice CT abdomen and chest with contrast revealed separate organs of both twins including both livers, which had separable blood supply, both pleurae touching each other through a defect in both sternums, and internal mammary vessels connected (Fig. 4).

#### 1.5.1 Anesthetic management

Pre-operative assessment of the twins' airways with no abnormalities. Two anesthesia teams and duplicate anesthetic resources were utilized. Before starting, atropine challenge test (iv atropine 0.12 mg) was performed to check the presence of shared circulation and it showed positive results. The induction of anesthesia was accomplished sequentially, starting with the one baby first. Inhalational induction of anesthesia was performed and intubation was performed without muscle relaxants using a 3 mm oral endotracheal tube. An assistant carefully lifted the second baby to position the first baby supine to facilitate endotracheal intubation and vice versa. Dextrose 5%- Ringer's lactate mixture 4 mL/kg was administered as maintenance fluid. Blood losses were calculated and replaced with blood transfusion. the baby twins were positioned on a warming water mattress.

*Except for duplicate anesthesia teams and logistic resources used in case no.4, the principles of anesthetic management and perioperative care were the same for all patients in this case series*.

#### 1.5.2 Operative procedure

##### 1.5.2.1 Positioning

the parts of the chest and abdomen facing surgeons were prepared as usual and the other parts prepared after holding the twins upwards.

##### 1.5.2.2 Operative steps

The operation was started by incision midway between the twins was done. The upper bony part of the chest was separated. Opened peritoneal cavities elucidated a small defect through which the transverse colon and gastric greater curvature of the right patient herniated into the left patient peritoneal cavity and both livers were fused in both ends of both left lobes. Separation of the fused livers was done exactly at their fusion line (the least vascular area) by coagulation diathermy and continuous suturing of the cut edges.

Re-examination of both patients as regards the position of abdominal viscera, the diaphragm which was almost intact with a small defect at the lower ends of both sternums with intact visible pleura. The defects in the sternums were left for spontaneous healing and skin over them was repaired. Both babies were transported intubated to the NICU.

The twins were unventilated after full recovery. Oral feeding started after 16 h. Both patients were discharged in the day 6th postoperative with the good general condition.

A small weak area in the wound site appeared 3 weeks after the operation a truss was advised for it.

## 2 Discussion

In epigastric heteropagus the parasite and autosite are attached in the epigastrium more in males, without bowel or bone connections and the lower extremities of the parasite are usually outside of the autosite [8] which agrees with our two cases of epigastric heteropagus.

Ventral parasites are supplied by the vessels of the falciform ligament, intercostal arteries, mesenteric arteries, epigastric arteries, left subclavian artery, and brachiocephalic trunk [9]. Omphalocele was reported in approximately half of epigastric heteropagus, but major omphalocele was seen only in a few cases [10,11]. Our two heteropagus cases had omphalocele major, one received blood supply from the internal thoracic vessels and the other from the falciform ligament. Because of the feasibility of the operation, we recommend the separation of the parasitic heteropagus as early as possible

Our reported omphalopagus had no associated anomalies. However, the proportion of associated malformations was 63%, without a specific association between certain types of conjoined twins and types of associated anomalies [11].

There is a significant female predominance in CT, particularly of the thoracopagus type and a significant male predominance in parapagus and parasitic types [11]. In accordance, our reported cases had the same sex predominance.

Mortality rates for twins who encountered in separation depending on the type of connection and the organs they share [12]. Recent advances in prenatal imaging, critical care, and anesthetic care have improved outcomes in separation surgery [9].

Up to date, The ideal age of separation surgery is a controversry. Separation surgery is an elective procedure done two to four months after birth. Sometimes an emergency separation may be needed if one of the twins dies, develops a life-threatening condition or threatens the survival of the other twin [12]. Spitz & Kiely preferred to operate at approximately three months of age, which allows time for detailed investigations to be conducted and enables separation to take place when the body wall can still rapidly expand to close substantial defects [12]. However, a high incidence of postoperative wound infection can occur, and separation is very harmful to the body's functional reserves. Therefore, Tannuri et al. preferred to operate at approximately 10 months of age, despite some psychosocial issues that may occur during the waiting period [12].

We faced two difficulties in our conjoined twin case 4, the first was that it is the 1st reported case in Upper Egypt to be operated in our institution and the second was the decision when to operate upon? Depending on our data and after reviewing related literature, we decided to operate on the conjoined twins as rapidly as possible because of difficult feeding and nursing care. Preoperative investigations provided adequate anatomic diagnosis and predicted the possibility of separation. To our knowledge, our patients are the youngest ever conjoined twins successfully separated. ”Lydia”, and ”Maya”; the youngest successfully separated CT in Switzerland in January 2016 were 8 days old; ours were younger than 7 days [11]. Although our twins had a shared liver, none had a complex or shared biliary tract. There were two independent hepatic circulations, and each twin had an inferior vena cava. Besides, we did not encounter any other anomaly and separation was possible and relatively easy.

concerning the repair of abdominal wall defect, it could be done anatomically or with mesh as Tanuri preferred [11]. In our conjoined twins, the simple anatomical repair was done.

Recently, using endoscopic minimally invasive techniques (laparoscopic and robotic surgery) in preoperative and operative

management can be applied for internal organ examination and if there are connections or not and can deal with connections. for external connections, open surgery is the mainstay of the separation technique.

## 3 Conclusion

Surgery to separate conjoined twins may range from very easy to very difficult depending on the point of attachment and the internal parts that are shared. In many cases, the surgery results in the death of one or both of the conjoined twins, particularly if they are joined at the head or share a vital organ and rectal manometry can be used only if anal canal affected or implicated during separation. We recommend the separation of conjoined twins as early as possible depending on the general condition of twins for achieving better outcome.

## Declaration of Competing Interest

The authors had reported no conflict of interest.

## Sources of funding

This study had not received any funding from any source.

## Ethical approval

The study has been approved from ethical committee of Faculty of medicine of Assiut University Approval Reference No. 17300170.

## Consent

Detailed written informed consent had been taken from patients, s parents of case No 1, case No 2, case No 3 and case No 4 for publishing this article with concern not to publish any personal details or names of the patients.

## Author contribution

1. Mohammed Hamada Takrouney: Writing - original draft.
2. Ibrahim Ali Ibrahim: Data curation.
3. Hala Saad Abdel-Ghaffar: Writing - original draft, Writing - review & editing.
4. Ahmed Ibrahim Abdel-Wahhab: Data analysis and collection.
5. Mahmoud Mohamed Mostafa: Conceptualization, Methodology.
6. Wesam Nashat Ali: Investigation, Formal analysis.
7. Mohamed Sayed Abd- Elaal: Investigation, Formal analysis.

## Registration of research studies

1. Name of the registry: Clinical Trial Government.
2. Unique identifying number or registration ID: NCT03388684.
3. Hyperlink to your specific registration (must be publicly accessible and will be checked): Clinical Trial Government NCT03388684.

## Guarantor

Ibrahim Ali Ibrahim: Professor in paediatric surgery department, faculty of medicine, Assiut university, Assiut, Egypt. Conduct of study, manuscript writing and preparation. Mobile Phone: +2 01005801291. E-mail address: Dr.ibrahimali@yahoo.com.

## Provenance and peer review

Not commissioned, externally peer-reviewed.
